# Clinical evaluation of a novel urinary protein competitive immunoassay in the detection of proteinuria in adults with sickle cell disease

**DOI:** 10.3389/fmed.2026.1787240

**Published:** 2026-09-01

**Authors:** Vincent Boima, Yvonne Dei-Adomako, Margaret Lartey, Akosua Asante-Offei, Markus Warlitz, Donald Very

**Affiliations:** 1 Department of Medicine & Therapeutics, University of Ghana Medical School, College of Health Sciences, Accra, Ghana; 2 Department of Haematology, University of Ghana Medical School, College of Health Sciences, Accra, Ghana; 3 Ghana Institute of Clinical Genetics, Korle Bu Teaching Hospital, Accra, Ghana; 4 The Institute for Life Sciences Collaboration, New Haven, CT, United States; 5 MS Degree Program in Biotechnology, College of Science and Engineering, Duquesne University, Pittsburgh, PA, United States

**Keywords:** early kidney disease detection, kidney disease, novel urinary protein, proteinuria, sickle cell disease

## Abstract

**Background:**

Sickle cell disease (SCD) affects millions of people worldwide and causes proteinuric kidney diseases. Early detection of proteinuria in patients with SCD would allow for earlier intervention, possibly limiting renal complications. Here, we report the results of a case-control, proof-of-concept study to assess the clinical performance of a novel urine protein immunoassay (UP immunoassay) for detecting small molecular weight proteins in the urine of patients with sickle disease (SCD) and healthy volunteers.

**Methods:**

This study recruited 100 patients with SCD (50 with proteinuria and 50 without) and 50 healthy controls without SCD or proteinuria. Patient demographic information and early morning urine specimens were collected. The samples were tested for proteinuria using the UP immunoassay and clinical laboratory tests for albumin and creatinine levels. Albumin: creatinine (ACR) and UP:creatinine (UPCR) ratios were calculated and compared across groups.

**Results:**

Of the 150 recruited participants, 147 completed the study. Our results demonstrated that the UPCR and ACR were substantially equivalent in the clinical detection of proteinuria across all patient groups. Despite this equivalence, the UPCR and ACR values did not correlate in the 147 patient samples tested (R^2^ = 0.5,113).

**Conclusion:**

UPCR measurement is clinically useful and equivalent to ACR measurement for detecting proteinuria across the three subject groups. The moderate correlation between the UPCR and ACR suggests that the UP immunoassay detects a collection of non-albumin proteins and peptides.

## Introduction

### Background

Sickle Cell Disease (SCD) is a group of inherited genetic disorders that are very common in sub-Saharan Africa (1–4). About 80% of all SCD cases occur in Sub-Saharan Africa (2), and it is estimated that 240,000 children are born with SCD each year (3). It has been projected that 404,200 babies with sickle cell disease will be born annually by the year 2050 (4, 5). With associated high morbidity and mortality (4), SCD is a cause of chronic kidney disease as manifested by proteinuria, an excess amount of protein in the urine (6). As standard tests for proteinuria measure albumin, a relatively large protein, we hypothesize that a test that measures smaller molecular weight proteins may provide an earlier indication of the onset of proteinuria, permitting earlier therapeutic intervention. Numerous studies in patients with chronic diseases have demonstrated the benefit of early therapeutic intervention in decreasing proteinuria and delaying the progression to end-stage kidney disease (7–9). Therefore, detection of proteinuria by a diagnostic test that detects smaller proteins and peptides than the traditionally used tests will permit earlier intervention, thereby delaying or preventing the onset of kidney disease, and may significantly improve the quality of life of patients with SCD in sub-Saharan Africa.

Previously, we developed, published, and patented an immunoassay utilizing proprietary reagents that can quantitate specific, small molecular weight (20–90 kDa), non-albumin, proteins, and peptides in human diabetic urine (10, 11). We have validated the use of this assay in the diagnosis of diabetic proteinuria (11), while its utility in detecting SCD-associated proteinuria is unknown.

Sickle cell nephropathy follows a recognizable natural history: kidney involvement typically begins with glomerular hyperfiltration, progresses through microalbuminuria and overt albuminuria, and may eventually culminate in a decline in estimated glomerular filtration rate (eGFR) and chronic kidney disease (12). Longitudinal cohort data indicate that albuminuria is frequently the earliest clinically detectable manifestation of this process and that its trajectory predicts subsequent renal decline (13). Because conventional screening (urine dipstick or albumin-based assays) is calibrated to detect albumin, a comparatively large (68 kDa) protein, these methods may be insensitive to early, lower-molecular-weight protein leakage that could precede measurable albuminuria. This raises the possibility that immunoassays capable of quantifying smaller, non-albumin urinary proteins could identify renal injury at an earlier stage than albumin-based testing alone, although this hypothesis has not previously been tested in an SCD population

Sickle cell disease (SCD) is a group of inherited genetic disorders that are associated with significant morbidity and mortality. This disease often adversely affects the glomerular kidney function, as manifested by an increased concentration of protein in the urine. Many studies in a wide variety of clinical conditions have demonstrated that early intervention to treat proteinuria can delay progression to chronic kidney disease and kidney failure. As current laboratory tests for proteinuria quantify albumin (a large 68 kDa protein), the use of a diagnostic test that quantifies smaller molecular weight urinary proteins may allow earlier detection and treatment of proteinuric kidney disease.

Despite the established prognostic value of albuminuria in SCD (14, 15) and the broader evidence that reducing proteinuria slows progression to end-stage kidney disease (16), no prior study has evaluated whether a non-albumin, small molecular weight urinary protein immunoassay can detect SCD-associated proteinuria, or how such a measure compares with the standard albumin-based reference method. The present study addresses this gap.

## Methods

### Study design

This was a case-control, proof-of-concept study involving prospectively collected urine samples from 150 normal non-SCD controls and patients with SCD (with and without proteinuria) attending the Sickle Cell Clinic at the Ghana Institute of Clinical Genetics, an adult Sickle Cell Clinic, Korle-Bu Teaching Hospital Accra, Ghana, and normal non SCD selected from volunteer blood donors visiting the Southern Zonal Blood Center (SZBC), Ghana.

### Participants

A total of 150 subjects [50 subjects, non-SCD controls [control], 50 patients, SCD-non-proteinuric [SCD-NP], and 50 patients with SCD-proteinuric [SCD-P]] were recruited for participation in this study. Two patients in the SCD-NP group and one patient in the SCD-P group withdrew their participation.

All the patients enrolled in this study met the inclusion and exclusion criteria. For SCD cases, the inclusion criteria were adults aged 18–55 years with results of hemoglobin electrophoresis clearly documented in their medical records and with or without a clinical diagnosis of proteinuria (defined as a urine dipstick result of at least 1 + for protein); in comparison, the inclusion criteria for normal controls were adults aged 18–55 years with no clinical evidence or history of SCD, kidney disease, or proteinuria. The exclusion criteria for both cases and controls were pregnant female subjects, smokers, history of recreational drug or alcohol abuse, current over-the-counter drug use, NSAID use within the last three months, acute illness at the time of enrollment, history of chronic illness, recent evidence or history of renal disease, and sickle cell crises within the last three months. All participants underwent blood testing to detect Human Immunodeficiency Virus (HIV), Hepatitis B Virus (HBV), and Hepatitis C (HCV) infections. Patients who tested positive were excluded from this study.

A structured questionnaire was used to collect participants’ sociodemographic and medical information after obtaining written consent. The participants’ identification number, highest level of education, employment status, profession, ethnicity, religion, marital status, age, sex assigned at birth, height, weight, and BMI were recorded. Additional clinical data included blood pressure (three measurements), urinary protein status, HBV status, HCV status, HIV status, number of vascular occlusive (VOC) events per year, blood hemoglobin (Hb) level, white blood cell (WBC) count, platelet count, serum sodium, potassium, chloride, bicarbonate, and creatinine, glomerular filtration rate (GFR), serum albumin, urinary albumin, urinary creatinine, and urinary albumin-to-creatinine ratio. The subjects enrolled in this study were age matched across all three clinical groups. The sex assigned at birth of the subjects across the three groups was slightly different, in that the control group contained slightly more males, whereas the SCD-NP and SCD-P groups contained more females. The demographic and mean serum chemistry differences observed between the three groups (Table 1) were likely due to either sex assigned at birth (e.g., weight and BMI) and/or clinical manifestation of disease (VOC per year, Hb, WBCs, and GFR).

**Table 1:** Description of patients enrolled in this clinical study.

Parameter	Controls (*n* = 50)	SCD-NP (*n* = 48)	SCD-P (*n* = 49)
**Demographics**			
Age, mean (years)	34.4	31.0	32.1
Age range (years)	21–51	18–55	18–55
Male, *n* (%)	28 (56%)	19 (40%)	20 (41%)
Female, *n* (%)	22 (44%)	29 (60%)	29 (59%)
**Anthropometrics**			
Weight (kg)*	73.5	62.5	59.0
Height (cm)	165.9	166.3	164.1
BMI (kg/m^2^)*	26.5	22.5	21.9
**Clinical Markers**			
VOC per year*	0	0–10	0–10
Hb (g/dL)*	13.4	8.7	8.1
WBCs (×10⁶/mL)*	5.5	8.8	12.7
Platelets (×10^3^/µL)	227.4	389.7	445.9
**Serum Chemistry**			
Sodium (mEq/L)	138.1	137.2	135.5
Potassium (mEq/L)	4.2	4.6	5.0
Chloride (mEq/L)	102.5	103.8	102.4
Bicarbonate (mEq/L)	26.7	23.1	23.2
Urea (mg/dL)	3.3	2.7	3.0
Creatinine (mg/mL)	68.9	51.1	52.4
GFR (mL/min/1.73m^2^)*	≥89	72–≥89	56–≥89
Albumin (g/L)	42.6	44.2	41.4

* Significant variations between the groups (*p* < 0.05). Sickle cell disease, non-proteinuric (SCD-NP) and sickle cell disease, proteinuric (SCD-P). Unless otherwise noted, data is presented as means. The units for each parameter are specified in parentheses. N, number of patients; BMI, body mass index; VOC, vascular occlusive events; Hb, hemoglobin; WBCs, white blood cell; GFR, glomerular filtration rate. “*”, indicates a parameter that varied significantly across groups.

### Test methods

Urine samples (5–10 mL) were collected from all the subjects. All urine samples were aliquoted in approximately 1·0 mL volumes and stored frozen at −80 °C until immunoassay analysis. One aliquot was stored at 4 °C for immediate testing of urinary albumin and creatinine levels at a hospital clinic. Urine albumin reagent was used to measure the albumin concentration using a turbidimetric method. In this test, anti-human serum albumin antibodies are combined with albumin from the sample to form immune complexes that scatter light in proportion to their size, shape, and concentration. The absorbance of these aggregates was proportional to the albumin concentration in the sample. The change in the absorbance was measured at 380 nm by subtracting the reference wavelength at 800 nm. To measure the urine creatinine concentration, creatinine forms a yellow-orange compound with picric acid in an alkaline medium. The rate of change in the absorbance at 520/800 nm was proportional to the creatinine concentration in the sample. The predicate albumin dipstick, URIT 10 V supplied by URIT Medical Electronic Co., Ltd., was used to determine urine protein. Participants with at least 1 + positive dipstick results were included in the SCD group with proteinuria, and SCD patients who tested negative were included in the SCD group without proteinuria.

Urinary dipsticks with ≥1 + proteinuria had a positive predictive value of 92% for urine protein excretion ≥300 mg in 24 h (12).

Aliquots of all patient urine samples were shipped to Green Mountain Antibodies, Inc. (Green Mountain, Burlington, VT, USA) for analysis using the competitive UP immunoassay. The assay was performed as previously described (11). All samples were analyzed in duplicate at dilutions of 1:2 and 1:24, as the endogenous concentration of UP was expected to differ greatly between the control, SCD-NP, and SCD-P patient groups. The UP results at each dilution were reported as the mean of duplicate results. Prior to shipment, samples were blinded to their identity and proteinuric status of the patient samples. For all samples, the albumin-to-creatinine ratio (ACR, mg/g) and UP-to-creatinine ratio (UPCR, mg/g) were determined and used in statistical analysis.

### Analyses

To evaluate the demographic equivalency of participants within all three clinical groups (Control, SCD-NP, and SCD-P), the number of patients, number of males, number of females, and the average height, weight, body mass index (BMI), and VOCs per year were determined for each group using EXCEL. Additionally, the circulating concentrations of Hb, WBCs, platelets, sodium, potassium, chloride, bicarbonate, urea, creatinine, albumin, GFR, urinary albumin, urinary creatinine, and urinary ACR in all patients were determined by the clinical laboratory at KBTH. The mean concentrations of these analytes within the clinical group were determined using EXCEL.

The ranges of ACR and UPCR values in control patient samples were determined by calculating the mean, median, 25th percentile, 50th percentile, 75th percentile, 95th percentile, and 97·5th percentile values. The percentiles were calculated using EXCEL.

To describe the characteristics of the outcome variables, analysis of variance (ANOVA; GraphPad) was used to assess the statistical difference in both the mean ACR and UPCR variances across the three study groups. Tukey's Multiple Comparison Test (GraphPad) was used to identify statistically significant differences in the group mean ACR and UPCR variances in the individual pairwise comparisons. In all analyses, a *p*-value ≤ 0·05 was deemed statistically significant.

Finally, to evaluate the concordance of ACR and UPCR values in all patients in this study, a linear regression correlation analysis of the sample analysis results was performed. The equation of the regression line and coefficient of correlation (R^2^) were calculated using EXCEL.

### Ethical considerations

This study was conducted in accordance with the ethical regulations of the University of Ghana College of Health Sciences, Ethical and Protocol Review Committee with protocol identification number of CHS-Et/M.3–P4.5/2020-2021 and Helsinki Declaration on Human Experiments in 1964 (revised in 2000).

Patient anonymity was ensured using codes and/or numeric identifiers to identify subjects.

Analysts performing sample analyses were blinded to the identity of the subjects and the subjects’ clinical groups. Access to patient data was restricted to researchers and interviewers.

Participants were fully informed of the nature of the study. They were also made aware that participation in this study was voluntary and that they were free to withdraw from the study at any time with no consequences. Subjects who agreed to participate in the study signed an Informed Consent Form.

## Results

Demographic characteristics and clinical chemistry profiles of the three groups of patients in this study are presented in Table 1.

The groups were similar in age (31–34 years), but there were more women in the SCD groups (59%–60% vs. 44%). Patients with SCD had lower weights (59–62 kg vs. 74 kg) and BMIs (22–23 vs. 27 kg/m^2^). In clinical terms, SCD markers were very different: haemoglobin ranged from 8.1–8.7 g/dL being lower in the SCD-P group compared to the SCD-NP and control groups; VOC episodes (0–10/year) occurring exclusively in SCD patients. GFR was lower in the SCD-P compared to the SCD-NP and control groups (>89 vs. 72–89 vs. 56 −89 mL/min/1.73m^2^), (*p* < 0.05).

Summary statistics were computed for the urine protein-to-creatinine ratio (UPCR) and the albumin-to-creatinine ratio (ACR) in the control group (*n* = 50). These statistics are reported in Table 2.

**Table 2:** Normal range analysis of control patients enrolled in this clinical study.

Statistic	UPCR (mg/g) in Control Subjects	ACR (mg/g) in Control Subjects
n	50	50
Mean	0·17	0·77
Median	0·12	0·50
25th %tile	0·10	0·30
50th %tile	0·12	0·50
75th %tile	0·19	0·75
95th %tile	0·43	2·47
97·5th %tile	0·49	3·21

The 97·5th percentile (percentile) of normal values, which is defined as the upper limit of normal values, for both the UPCR and ACR in normal subjects, are indicated.

The mean for UPCR (mg/g) was 0.17, the median was 0.12, and the 25th, 50th, and 75th percentiles were 0.10, 0.12, and 0.19, respectively. The 95th percentile was 0.43 and the 97.5th percentile was 0.49. The mean for ACR (mg/g) was 0.77, the median was 0.50, the 25th, 50th, and 75th percentiles were 0.30, 0.50, and 0.75, and the 95th and 97.5th percentiles were 2.47 and 3.21, respectively*.*

### UPCR and ACR values in control and SCD patient groups

**Figure 1:**
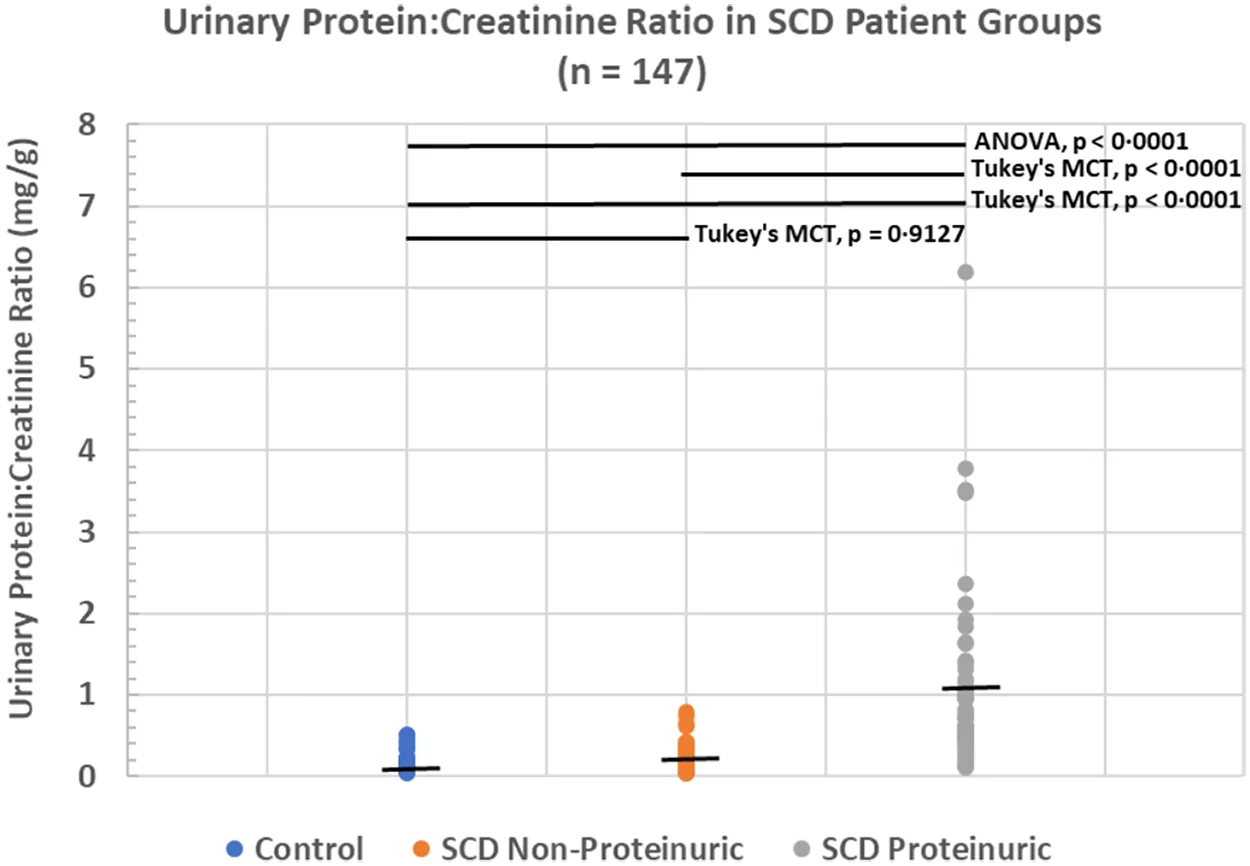
Determination of the urinary protein to creatinine ratio in control and SCD patient urine samples clinically differentiates proteinuric from non-proteinuric patients. The statistical significance of the differences in the sample group means was assessed using an Analysis of Variance (ANOVA, *α* = 0·05). Pairwise comparisons of the group means were performed using Tukey's multiple comparison test (MCT). Each dot in the graph represents an individual patient. The small horizontal lines indicate the group means. The large horizontal lines at the top of the graph illustrate the individual group means compared using ANOVA or Tukey's MCT.

**Figure 2:**
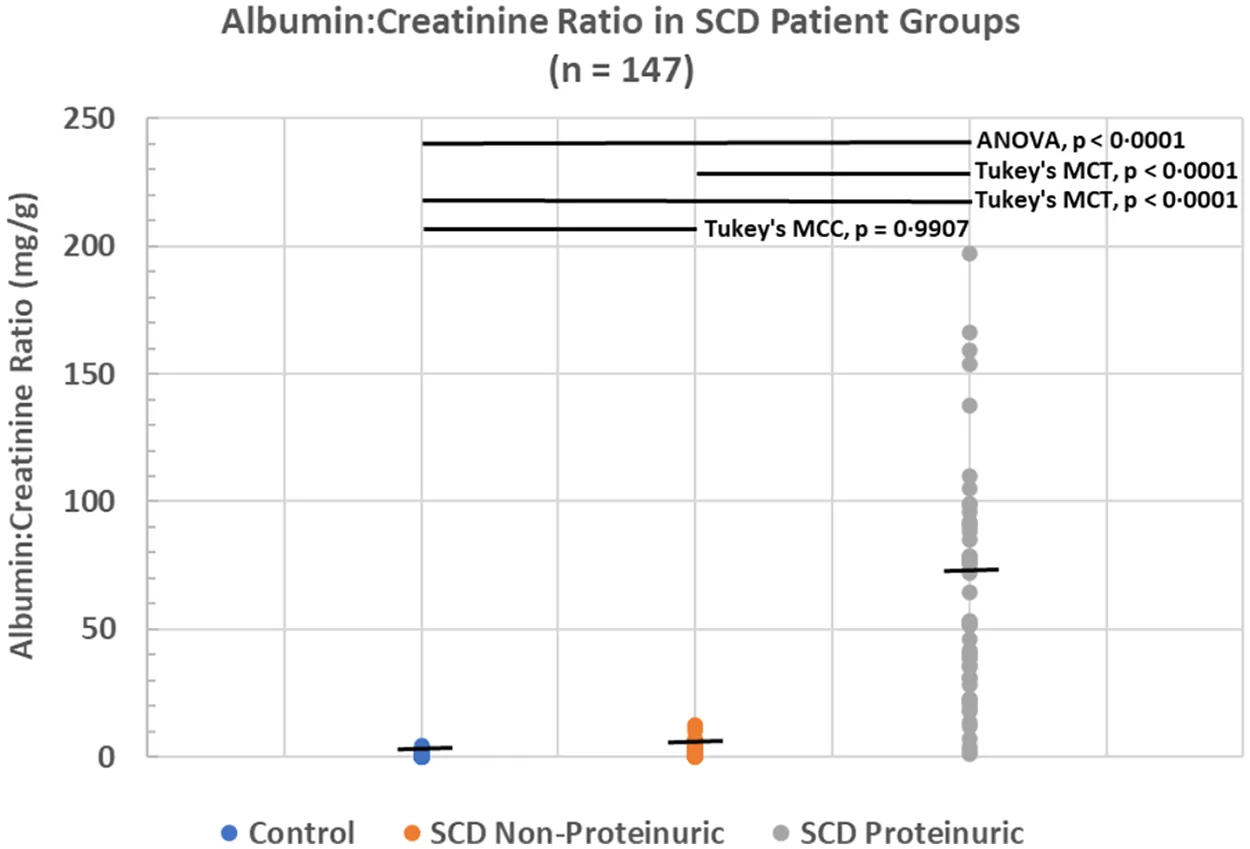
Determination of the albumin to creatinine ratio in control and SCD patient urine samples clinically differentiates proteinuric from non-proteinuric patients. The statistical significance of the differences in the sample group means was assessed using an Analysis of Variance (ANOVA, *α* = 0·05). Pairwise comparisons of the group means were performed using Tukey's multiple comparison test (MCT). Each dot in the graph represents an individual patient. The small horizontal lines indicate the group means. The large horizontal lines at the top of the graph illustrate the individual group variances compared using ANOVA or Tukey's MCT.

Figures 1, 2 present statistical comparisons of the mean UPCR and ACR values across the three patient groups in this study. The results depicted in Figure 1 for UPCR demonstrate the clinical utility of the urinary protein immunoassay in identifying proteinuria in patients with SCD. Notably, UPCR was not useful for distinguishing between the Control and SCD-NP groups (*p* < 0·9,127, *α* = 0·05). Combined with the results depicted in Figure 2, these results demonstrate that the UPCR measurement is substantially equivalent to the reference clinical method (ACR measurement) in differentiating proteinuric from non-proteinuric SCD patients.

### UPCR and ACR values in SCD patient and control sample groups showed moderate correlation

**Figure 3:**
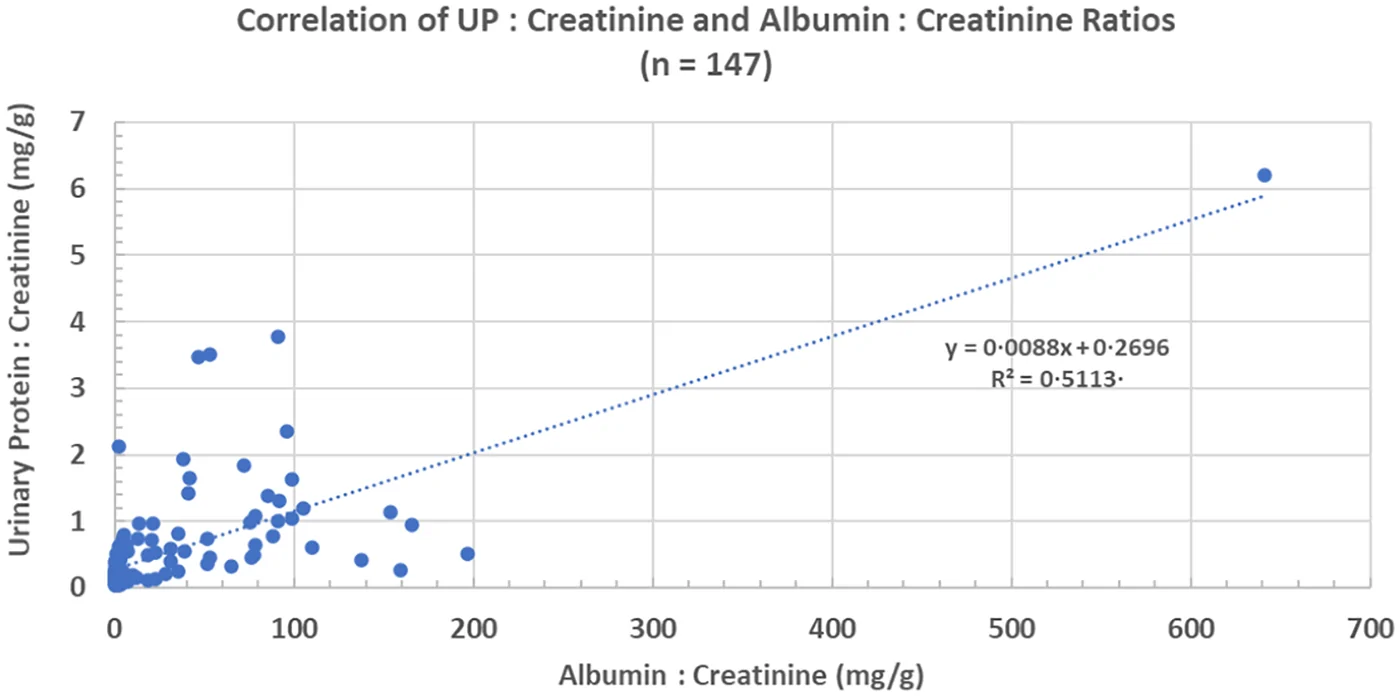
Linear regression analysis of ACR and UPCR values in All 147 study samples demonstrates that the ACR and UPCR values do not correlate. Subject samples from all three clinical groups were evaluated for ACR and UPCR as previously described.

We have previously demonstrated that the UP immunoassay quantifies a specific collection of small molecular weight, non-albumin, and urinary proteins/peptides (11). If this is true, it would be expected that, despite their substantial equivalence in the discrimination of normal, non-proteinuric, and proteinuric patient populations, UPCR and ACR values would show only little to moderate correlation, as the two tests detect different urinary proteins that are subjected to differential glomerular filtration and microtubule reabsorption rates. To test this hypothesis, the UPCR and ACR values in all 147 study samples were correlated using linear regression analysis and the correlation coefficient (R^2^) was calculated. The results of the correlation analysis are shown in Figure 3.

The results presented in Figure 3 demonstrate that in this collection of 147 clinical urine specimens, the ACR and UPCR values showed moderate correlation (R^2^ = 0.5113). This supports our hypothesis that, despite the clinical equivalence of ACR and UPCR measurements in detecting proteinuria, the UP immunoassay detects a collection of proteins/peptides that are different from albumin.

## Discussion

This proof-of-concept study validates the urinary protein (UP) immunoassay (and its proprietary reagents) as a clinically effective method for identifying SCD-associated proteinuria, demonstrating significant equivalence between UPCR and ACR in distinguishing proteinuric from non-proteinuric individuals within the study cohort (groupwise ANOVA/Tukey comparisons as detailed in the study).

The UP immunoassay is designed to measure a certain group of non-albumin urinary proteins and peptides. This is in line with the general clinical idea that the protein-to-creatinine ratio (PCR) measures the total amount of urinary protein (including non-albumin proteins), while albumin-focused tests (ACR or albumin dipsticks) only show albuminuria and may not show other types of protein (12). KDIGO's current guidelines stress that albumin reagent strips might miss non-albumin proteins and suggest that positive semiquantitative dipstick results be confirmed with quantitative laboratory testing, which is reported as ACR or PCR (14).

Since albumin is a relatively large protein and ACR specifically measures albumin excretion, an assay that detects non-albumin (often lower-molecular-weight) proteins may reveal a distinct phenotype of kidney injury compared to ACR alone, potentially encompassing earlier or more tubular-predominant protein handling abnormalities. This is biologically plausible in sickle cell nephropathy, where kidney involvement typically starts with hyperfiltration and progressive glomerular/tubular injury, subsequently leading to albuminuria and eventual decline in estimated glomerular filtration rate (eGFR) (15).

Clinically, the justification for prioritizing earlier detection is substantiated by longitudinal SCD cohorts indicating that albuminuria is an initial manifestation and that elevated or sustained albuminuria forecasts accelerated eGFR decline and the onset of CKD (13). Consequently, a scalable assay that detects albumin-independent urinary protein signals may serve as a beneficial supplementary screening tool, especially if forthcoming prospective studies reveal additional prognostic significance beyond ACR, which is the preferred and standardized marker for CKD staging and risk stratification in most contexts (14).

Proteinuria is prevalent among adults with sickle cell disease (SCD) and appears to be more pronounced in severe genotypes (HbSS vs. HbSC). A multicenter cohort utilising KDIGO definitions reported chronic kidney disease (CKD) in 39.8% of adults with SCD (17). In this cohort, a higher KDIGO UACR category independently predicted adverse outcomes during follow-up, including an approximately two-fold increased risk of CKD progression and an approximately 1.8-fold increased risk of mortality (17), thereby underscoring the clinical significance of urinary protein/albumin excretion as a risk-stratification marker in SCD.

In kidney disease populations, proteinuria/albuminuria is a well-established prognostic marker and a modifiable treatment target. In a comprehensive individual participant-level consortium meta-analysis of 28 cohorts (*n* = 675,904; 7,461 ESKD events), a 30% reduction in ACR during a baseline period correlated with a diminished subsequent risk of ESKD (adjusted HR 0.83; 95% CI 0.74–0.94), thereby endorsing the utilization of albuminuria change as a clinically significant surrogate for long-term renal outcomes (16).

Adults with chronic kidney disease (CKD) and clinically significant albuminuria should receive kidney-protective therapy aimed at decreasing albuminuria/proteinuria. This includes renin–angiotensin system inhibition (ACE inhibitor or ARB) when albuminuria is present, and SGLT2 inhibitor therapy for eligible adults with CKD, regardless of diabetes status in specified risk groups, to mitigate the risk of CKD progression and cardiovascular events (14).

Therapeutic trials and guideline syntheses endorse the “proteinuria as target” paradigm: RAS blockade has shown renal benefits in proteinuria chronic kidney disease populations, supported by evidence compiled in KDIGO blood pressure guidelines, referencing trials such as REIN and AASK. KDIGO's most recent CKD guidance also says that SGLT2 inhibitors lower the risk of kidney disease getting worse in adults with CKD, and that this is true for both people with and without diabetes (14).

In summary, these data highlight clinical justification for the prompt detection of renal injury in SCD, emphasizing the potential utility of quantifying urinary protein signals that may elude albumin-centric testing, to facilitate the timely commencement of established nephroprotective therapies and consequently reduce the advancement to severe CKD.

This study was designed as a preliminary clinical feasibility assessment of a novel urinary protein (UP)immunoassay and its derived urine protein-to-creatinine ratio (UPCR). The limited number of participants in each clinical category restricts statistical power, particularly for estimating reference limits and conducting robust subgroup comparisons. Consequently, larger, adequately powered case–control studies are required to validate diagnostic performance and reproducibility. Again, future studies should compare UPCR with KIM-1 and Cystatin C measurements in parallel, to determine whether the UP-immunoassay signal correlates with, complements, or is independent of these established injury markers. Furthermore, parallel measurement of sEPCR alongside UPCR in future cohorts could help determine whether elevated UPCR partly reflects endothelial injury markers shed into urine, which would support the biological plausibility of UPCR as an early, mechanistically distinct signal in SCD nephropathy.

Furthermore, the current data do not demonstrate that UPCR facilitates the earlier detection of incident proteinuria; instead, this remains a biologically plausible hypothesis necessitating prospective validation. Longitudinal cohorts in SCD show that kidney involvement changes over time, usually starting with hyperfiltration and then albuminuria. They also show that repeated measurements of albuminuria are important for predicting the progression of CKD and mortality. This shows how important it is to do serial urine sampling and follow up on outcomes in future validation work (15, 17)

Consequently, the early-detection hypothesis must be evaluated in adequately powered, longitudinal studies featuring standardized, repeated urine collection among both controls and SCD participants, prior to and following the emergence of clinically defined proteinuria/albuminuria, with endpoints encompassing persistence/progression and deterioration of kidney function.

In conclusion, the study supports UPCR (through a non-albumin immunoassay) as an albumin-independent marker that corresponds with ACR for proteinuria classification at the group level, while assessing a biologically unique urinary peptides, necessitating additional prospective evaluation before clinical implementation as a standalone screening test.

## Data Availability

The raw data supporting the conclusions of this article will be made available by the authors, without undue reservation.
